# The Crisis Close at Hand: How COVID-19 Challenges Long-Term Care Planning for Adults with Intellectual Disability

**DOI:** 10.1089/heq.2020.0020

**Published:** 2020-06-09

**Authors:** Charmaine Wright, Caren Steinway, Sophie Jan

**Affiliations:** ^1^ Center for Special Health Care Needs, Christiana Care Health System, Wilmington, Delaware, USA.; ^2^ Department of General Pediatrics, Northwell Health, Hempstead, New York, USA.; ^3^ Department of Pediatrics, Donald and Barbara Zucker School of Medicine at Hofstra/Northwell Health, Hempstead, New York, USA.

**Keywords:** disability, COVID-19, long-term care planning

## Abstract

Whether cared for in the community or in a facility, adults with intellectual disability are among the most vulnerable individuals in the United States. Families caring for these individuals face financial, social, and emotional stress as they navigate long-term care choices for their loved ones. COVID-19 has stressed an already overwhelmed and disparate system.

## Introduction

With 23.2 million Americans living with a disability that affects cognitive function, COVID-19 hastens an impending public and personal crisis.^1^ For families caring for their adult loved ones with intellectual disability at home, a bewildering patchwork of services usually provides caregiver respite and familial financial stability. At baseline, there are challenges to this system now stressed by the pandemic. In the past few weeks, we have seen the uptick of families needing caregiver support for anxiety and uncertainty as day programs were closed and stay at home orders were enforced.

In a panic, a mother called 911 yesterday at 10 a.m. due to her inability to calm her adult child with autism who is no longer on his schedule of day program and work that helped manage his anxiety, aggression, and impulsivity. We have also seen the uptick of family members asking for Family and Medical Leave Act or other financial support to stay home to prevent their relative from contracting COVID-19. I wrote in a letter to an employer yesterday, that the added risk of a respiratory illness in a patient with cerebral palsy and weak cough, for example, can be deadly.

By 3 p.m. also yesterday, we were called by a 92-year-old mother who serves as the primary caregiver for her adult daughter with intellectual disability. Her daughter has a rare congenital disease that caused the inability to talk and walk, and in the past year she developed other diseases of aging, including hypertension and diabetes. Her mom was still well enough to be able to call us from the COVID unit in our hospital, desperately begging for the care of her daughter who remained at home. Their plan for her did not include an acute illness such as COVID-19. Although multiple conversations had occurred regarding what would happen when she died, her daughter remained on a wait list for a group home. With many residential facilities currently closed to new admissions, yesterday we began the arduous process of emergency placement in the time of COVID-19.

Emergency placement is defined as unexpectedly needing residential long-term care in a nursing home or facility. It is our practice's mission to avoid this emergent need by carefully planning with and then supporting a family's strategy for future care. This may include community or residential living and depend on siblings or unrelated guardians. Widespread knowledge on how to navigate these decisions simply does not exist. Add an unexpected crisis such as COVID-19 and you have the perfect storm of yesterday—caregivers and patients in potential danger as they shelter in place, caregivers unable to work, and family members with intellectual disability left alone at home without any support and unable to use the myriad of virtual touchpoints that allow connections between those not physically together. Each state has a different way of supporting these individuals and their families, and funding is highly disparate across the country; so too has the response been to families who live in this current crisis.

And what about those individuals with intellectual disability who already reside in a group home or facility? They and their families are not immune to a stressed system. In fact, group homes and facilities began and continue to be hotbeds of COVID-19 infection. For several weeks, residential dwellings have limited visitors, volunteers, and vendors while promoting social distancing and struggling to keep an infection-free staff. In Delaware, yesterday's executive order by the governor focused on long-term care facilities and their ability to cohort staff and patients with COVID-19 to prevent spread.^2^ As dynamic plans are implemented that involve potential moves of residents to a hospital or another facility, the inability to visit or even communicate with busy staff has left many families without any information on the status of their loved ones.

With the curve flattening in many areas, we must turn to glimmers of hope and work to be a part of the light. There are initiatives afoot to implement tools that help families plan for the future before and during any crisis. S.J.'s team from Northwell Health is working to develop and test a website that helps families consider the important factors and document and communicate future long-term plans for loved ones with intellectual disability. Initiatives to ensure the use of simple communication devices in hospital settings such as baby monitors and Amazon Alexa can help families separated by distance assist in speaking for their loved one. This Saturday, Delaware's Division of Developmental Disabilities Services (DDDS) will host a second Zoom meeting to provide information to families during this unprecedented time and a hotline to help families navigate programs and choices. It is our choice right now as a country, to decide how we will treat the most vulnerable among us. With cautious optimism, we must infuse the support systems of those who may have the least among us, with the most.

## Abbreviations Used

DDDS: Delaware's Division of Developmental Disabilities Services
